# Resting and Initial Beta Amplitudes Predict Learning Ability in Beta/Theta Ratio Neurofeedback Training in Healthy Young Adults

**DOI:** 10.3389/fnhum.2015.00677

**Published:** 2015-12-21

**Authors:** Wenya Nan, Feng Wan, Mang I Vai, Agostinho C. Da Rosa

**Affiliations:** ^1^Department of Electrical and Computer Engineering, Faculty of Science and Technology, University of MacauMacau, China; ^2^Department of Bio Engineering, Instituto Superior Tecnico and Systems and Robotics Institute, University of LisbonLisbon, Portugal

**Keywords:** neurofeedback training, beta/theta ratio, self-regulation, prediction, learning indices

## Abstract

Neurofeedback (NF) training has been proved beneficial in cognitive and behavioral performance improvement in healthy individuals. Unfortunately, the NF learning ability shows large individual difference and in a number of NF studies there are even some non-learners who cannot successfully self-regulate their brain activity by NF. This study aimed to find out the neurophysiological predictor of the learning ability in up-regulating beta-1 (15–18 Hz)/theta (4–7 Hz) ratio (BTR) training in healthy young adults. Eighteen volunteers finished five training sessions in successive 5 days. We found that low beta (12–15 Hz) amplitude in a 1-min eyes-open resting baseline measured before training and the beta-1 amplitude in the first training block with 4.5-min duration could predict the BTR learning ability across sessions. The results provide a low cost, convenient and easy way to predict the learning ability in up-regulating BTR training, and would be helpful in avoiding potential frustration and adjusting training protocol for the participants with poor learning ability.

## Introduction

Neurofeedback (NF) training enables people to learn self-regulating their brain activity and in doing so potentially improve their behavior or cognitive performance (Dempster and Vernon, 2009). Numerous studies have shown the NF benefits on enhancement of cognitive and behavioral performance (Vernon et al., 2003; Ros et al., 2009, 2014; Nan et al., 2012, 2013; Enriquez-Geppert et al., 2014a; Gruzelier, 2014a; Mottaz et al., 2015) as well as treatment of a wide variety of neurological and psychiatric disorders such as attention-deficit/hyperactivity disorder (ADHD; Arns et al., 2009, 2014), autistic spectrum disorder (Coben et al., 2010) and major depressive disorder (Choi et al., 2011; Peeters et al., 2014; Cheon et al., 2015).

Neurofeedback learning ability, which indicates how well the training individuals learn to self-regulate their EEG pattern, is critical in NF training, since it helps to understand the NF process and optimize the NF protocol (Gruzelier, 2014b; Zuberer et al., 2015). Moreover, it has crucial mediation link with the enhancement of behavior or health after training (Gruzelier, 2014a). For sensorimotor rhythm (SMR) NF, Schabus et al. (2014) performed 10 training sessions to up-regulate the amplitude of SMR (12–15 Hz) in a population of young primary insomnia patients for the purpose of enhancing their sleep quality and memory performance, and the results found significant inter-individual positive correlations between SMR learning and the change in overnight memory consolidation and increased fast non-rapid eye movement (NREM) sleep spindles; Ros et al. (2009) reported a significant positive correlation between SMR learning and enhancement of surgical skills following SMR training. In alpha NF, the enhancement in short term memory was positively related to upper alpha learning (Nan et al., 2012). In theta/alpha ratio training, the theta/alpha ratio learning had high correlation with musical performance improvement (Egner and Gruzelier, 2003). To sum up, NF learning plays an important role in training efficiency.

However, learning ability varies among training individuals and even a high percentage of non-learners (i.e., participants cannot achieve successful self-regulation) have been reported in many training protocols (Kotchoubey et al., 1999; Hanslmayr et al., 2005; Kropotov et al., 2005; Doehnert et al., 2008; Weber et al., 2011; Zoefel et al., 2011; Kouijzer et al., 2013; Dekker et al., 2014; Enriquez-Geppert et al., 2014a; Schabus et al., 2014; Quaedflieg et al., 2015; Reichert et al., 2015). This severely affects NF training efficiency and hinders the application and further development of NF training. To overcome this difficulty, the identification of early predictors for NF learning is a vital step. It would be helpful to prevent potential frustration and expensive training sessions, save cost on non-learners, design and modify the training protocol accordingly, and understand the reason of poor NF learning ability.

Some recent studies have identified predictors of NF learning for several NF protocols. The learning predictors in SMR NF include initial training performance in early sessions (Weber et al., 2011), control belief (Witte et al., 2013), resting SMR activity (Reichert et al., 2015), and morphology of brain structures (Ninaus et al., 2015). Regarding gamma NF, the learning ability can be predicted by gray matter volumes in the supplementary motor area and left middle frontal gyrus (Ninaus et al., 2015). For frontal-midline theta NF, the morphology of brain structures predicts the NF learning success (Enriquez-Geppert et al., 2013). Our previous work has reported that resting alpha activity predicts the NF learning in alpha NF (Wan et al., 2014). In summary, the NF learning predictors from the literature include the psychological parameters such as control belief and neurophysiological parameters such as resting and initial EEG activity and the morphology of brain structures, which may depend on the training protocols. Nevertheless, the research in prediction of NF learning is still at its early stage.

In various NF protocols, the enhancement of beta-1 (15–18 Hz) to theta (4–7 Hz) ratio (BTR) by NF training at different electrode locations has shown promise as a potential treatment in ADHD (Bakhshayesh et al., 2011; Duric et al., 2012; Lofthouse et al., 2012), reading disabilities (Sadeghi and Nazari, 2015), and physical balance problems in different diseases (Hammond, 2005; Azarpaikan et al., 2014). Besides clinical treatments, BTR training at Cz has been reported to enhance arousal level (Egner and Gruzelier, 2004) and response speed (Studer et al., 2014) in healthy people. Nonetheless, some studies also reported non-learners in this training protocol (e.g., Studer et al., 2014). The prediction of BTR NF learning, however, has remained unanswered so far.

This study therefore aimed to find out the predictor of learning ability in BTR NF on healthy young adults from neurophysiological variables. Considering that BTR NF using the bipolar montage of two electrodes directly under O1 and O2 has shown benefits in physical balance and visual-spatial attention ability in patients (Hammond, 2005; Azarpaikan et al., 2014; Sadeghi and Nazari, 2015) and it has potential for peak performance training in areas such as gymnastics or ballet (Hammond, 2005), the training was performed on the above location by bipolar montage. Eighteen healthy young adults performed one training session per day for five sessions totally. In order to predict the NF learning as early as possible, the EEG activities measured before training and in the initial training were taken into consideration.

## Materials and Methods

### Participants

Eighteen healthy volunteers (eight females) finished all NF training procedure. The age of the participants ranged from 19 to 29 years-old (mean = 24.33; *SD* = 2.63). Inclusion criteria for the NF training were as follows: no history of psychiatric or neurological disorders, no psychotropic medications or addiction drugs, and with normal or corrected-to normal vision. Prior to the experiment, a written informed consent was obtained from all participants after the experimental nature and procedure were interpreted and their questions were answered. After experiment, all participants received monetary compensation for their participation. The protocol was in accordance with the Declaration of Helsinki and approved by the Research Ethics Committee (University of Macau).

### NF Training

This study employed the BTR training protocol proposed by Hammond (2005) for physical balance enhancement. A bipolar montage was used by two electrodes directly under electrode sites O1 and O2 and barely above the inion, where is approximately over visual processing areas involving in analysis of movement, position, orientation, and depth (Hammond, 2005). Furthermore, function improvement in the vicinity of primary visual cortex may improve the visual guidance for the cerebellum (Hammond, 2005). Thus, the same training protocol was employed in the current work. A ground electrode was placed on the forehead. The EEG signal was amplified by an EEG amplifier (Vertex 823 from Meditron Electomedicina Ltd, SP, Brazil) with an analog band-pass filter from 0.1 to 70 Hz and recorded by a Somnium system (Cognitron, SP, Brazil) at a sampling frequency of 256 Hz. In the Somnium system, the signals were filtered by a band-pass filter from 0.5 to 30 Hz, and a notch filter at 50 Hz. The impedance was maintained below 10 kΩ for all electrodes.

The training feature was set to the beta-1 amplitude to theta amplitude ratio and presented to the subjects in visual format. Using the amplitude spectrum instead of the power spectrum prevents excessive skewing which results from squaring the amplitude, and thus increases statistical validity (Sterman and Egner, 2006). The amplitude was calculated by fast Fourier-transforms (FFTs) every 125 ms with a 2-s data window. Thus, the frequency resolution was 0.5 Hz.

Each participant received one training session per day for a total of five sessions in five consecutive days. Each session consisted of five training blocks, and each block had four 1-min trials and between each two consecutive trials there was an interval of 10 s. Thus, each session had a training duration of 20 min totally. After each training block, the participants could have a rest and they were required to write down the mental strategy in each trial. Two 30-s epochs with eyes open and two 30-s epochs with eyes closed resting baseline were recorded before and after each session, which were named as pre baseline and post baseline respectively. Thus, there were seven periods in each training day including pre baseline, Block 1, Block 2, Block 3, Block 4, Block 5, and post baseline.

The feedback display contained two 3D objects: a sphere and a cube. The sphere radius reflected the feedback parameter in real time and if this value reached a threshold (Goal 1) the sphere color changed. This sphere was made of several slices and the more slices it had, the smoother it looked. The cube height was related to the period of time for which Goal 1 kept being achieved continuously. If Goal 1 was being achieved continuously for more than a predefined period of time (2 s), Goal 2 was accomplished and the cube rose up until Goal 1 stopped being achieved. Then the cube started falling slowly until it reached the bottom or Goal 2 was achieved again (Nan et al., 2012). Therefore, the participants were instructed to apply mental strategies to increase the sphere size or keep the cube as high as possible. No instructions about the effective mental strategies were given since the effective mental strategies vary across individuals (Nan et al., 2012).

In the first block of each session, the feedback threshold was empirically set to 90% of the BTR in pre baseline of the corresponding session, in order to have a proper difficulty level for the subject. After each block, we calculated the percentage of time for the training parameter above threshold in the training block. If the percentage of time was above 70%, the threshold would be increased by 0.1 in the next block.

### Data Analyses

#### EEG Amplitude Calculation

Absolute EEG amplitude has large individual difference owing to influences of many factors (such as anatomical and neurophysiological properties of the brain, cranial bone structure, and electrode impedances; Kropotov, 2009). Hence, relative amplitude was calculated in order to ensure comparability across participants (Reichert et al., 2015). The relative amplitude was defined to the analyzed frequency band amplitude relative to the EEG band amplitude from 4 to 30 Hz. The analyzed frequency bands including theta (4–7 Hz), alpha (8–12 Hz), low beta (12–15 Hz), and beta-1 (15–18 Hz) bands. The relative amplitude of these frequency bands were calculated for all resting baseline and training trials according to Eq. 1 where the *High* and the *Low* were the high and low boundaries of each frequency band and *X*(*k*) was the frequency amplitude spectrum calculated by FFT. The relative amplitude in each training block was the average of four training trials in the block, and the average of five training blocks in each session was taken as the session relative amplitude.

(1) $$relative\;\;amplitude=\frac{\frac{\sum_{k=Low}^{High}\;X(k)}{High-Low}}{\frac{\sum_{k=4}^{30}\;X(k)}{30-4}}\,\;\;\;\;\;\;\;\;\;\;\;\;\;\;\;\;\;\;\;\;\;\;\;\;\;\;\;\;\;\;\;\;\;\;\;\;\;(1)$$

#### NF Training Effects on EEG Activity

The NF training effects on EEG activity are usually examined by within training sessions compared to baseline and across sessions (Dempster and Vernon, 2009; Enriquez-Geppert et al., 2014b; Wan et al., 2014). Repeated measures analysis of variance (ANOVA) were performed not only in the BTR, beta-1, and theta but also their neighboring frequency bands alpha and low beta. For all statistical analyses, in cases of sphericity violations, Greenhouse–Geisser corrections were applied. Regarding the within sessions compared to baseline analysis, the within-subject factor was Period (seven levels: pre baseline, Block 1, Block 2, Block 3, Block 4, Block 5, post baseline). For the across sessions, the within-subject factor was Session (five levels: Session 1, Session 2, Session 3, Session 4, Session 5). Additionally, the training independence (i.e., whether the training has effect on other bands) proposed by Zoefel et al. (2011) was examined by the alpha and low beta changes across sessions.

#### NF Learning Assessment and Prediction

Here, the learning ability was assessed by two indices. One was the average within-session change calculated by Eq. 2 where *k* was the session number, *j* was the block number, *n* was total number of sessions, and *m* was the total number of blocks. L1 described the average learning ability in short term (Wan et al., 2014). Another learning index L2 was the linear regression slope of BTR value over 5 sessions, which presented the learning ability across whole training process and indicated accumulative training effects.

(2) $$L_{1}=\frac{\sum_{k=1}^{n}\sum_{j=2}^{m}\left(block\,j-block\,\;1\,\;\;of\,\;\;k^{th}\,\;\;session\right)}{n}\,\;\;\;\;\;\;\;\;\;\;\;\;(2)$$

We defined the learners and non-learners according to L1 and L2, respectively, since the two indices indicated the learning from different aspects. Based on L1, the subject who had positive value in L1 was defined as learner_L1 (i.e., the subject was able to enhance BTR within sessions), while the subject with negative L1 was defined as non-learner_L1. Similarly, the subject who had positive value in L2 was defined as learner_L2 (i.e., the subject was able to enhance BTR across sessions), while the subject with negative L2 was defined as non-learner_L2.

All data were normally distributed examined by the Shapiro–Wilk test. By the adjusted box-plot rule for outlier detection (Pernet et al., 2013), one subject’s beta-1 in Block 1 of Session 1 was outlier (this subject was learner_L1 but non-learner_L2), and two subjects’ theta in the eye-open baseline before NF were outliers (the two subjects were both learner_L1 and learner_L2). In order to achieve reliable statistical results, the outliers were deleted from the corresponding feature in the following analyses. Independent *t-*test was used to find out the significant discriminative features between learners and non-learners from all analyzed frequency bands measured in pre baseline before Session 1 and Block 1 in Session 1. In order to predict the NF learner and non-learner, step-wise linear discriminant analyses (LDA) were employed. Inputs of the LDA were the significant discriminative features recognized by independent *t-*test.

## Results

### NF Training Effects on EEG Activity

#### Within Sessions Compared to Baseline

**FIGURE 1:**
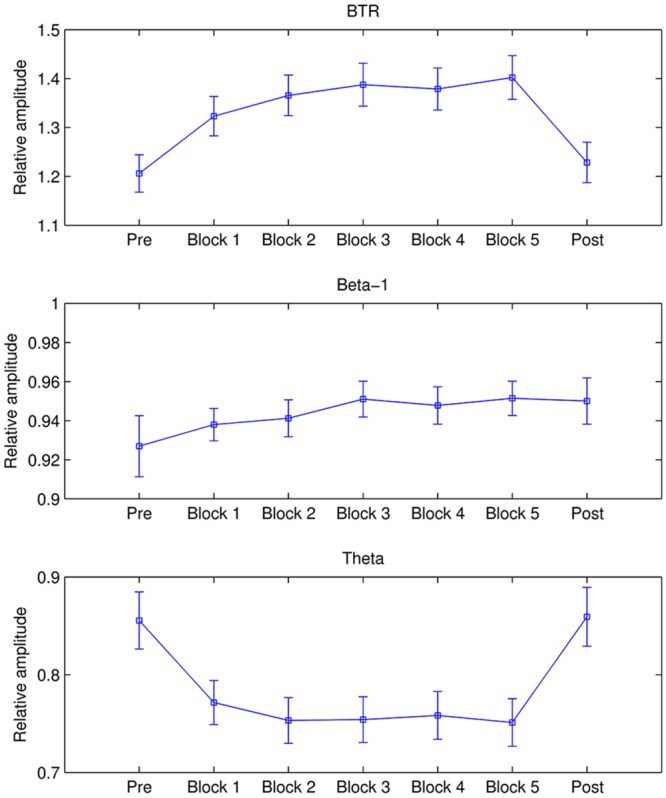
**Mean BTR, beta-1, and theta in each period.** The error bars depict standard error of the mean (SEM).

The mean beta-1, theta and their ratio in each period across all participants are shown in **Figure 1**. It is observed that beta-1 and BTR in all training blocks are higher than pre baseline whereas theta in all training blocks are lower than pre and post baseline. A repeated measures ANOVA showed a significant main effect of Period in BTR [*F*(4.361,388.17) = 15.752, *p* < 0.001, $\eta_{p}^{2}$ = 0.15] and theta [*F*(3.815,339.526) = 13.582, *p* < 0.001, $\eta_{p}^{2}$ = 0.132] but not in beta-1. From further pairwise comparisons using the Bonferroni correction, BTR in all training blocks significantly increased compared to pre baseline (*p* < 0.001) while Blocks 2–4 were significantly higher than post baseline (*p* < 0.01). Similarly, theta significantly decreased in all training blocks compared to pre and post baseline (*p* < 0.01).

Additionally, alpha decreased from pre baseline to Block 5 and then rebounded in post baseline, whereas low beta was higher in five training blocks compared to pre and post baseline. Repeated ANOVA found significant difference between periods in alpha band [*F*(3.458,307.729) = 6.244, *p* < 0.001, $\eta_{p}^{2}$ = 0.066] and low beta [*F*(4.38,389.834) = 2.441, *p* = 0.041, $\eta_{p}^{2}$ = 0.027]. Pairwise comparisons with the Bonferroni correction revealed that alpha in pre baseline was significantly higher than in Block 3 (*p* = 0.01), Block 4 (*p* = 0.026), and Block 5 (*p* = 0.017).

#### Across Sessions

**FIGURE 2:**
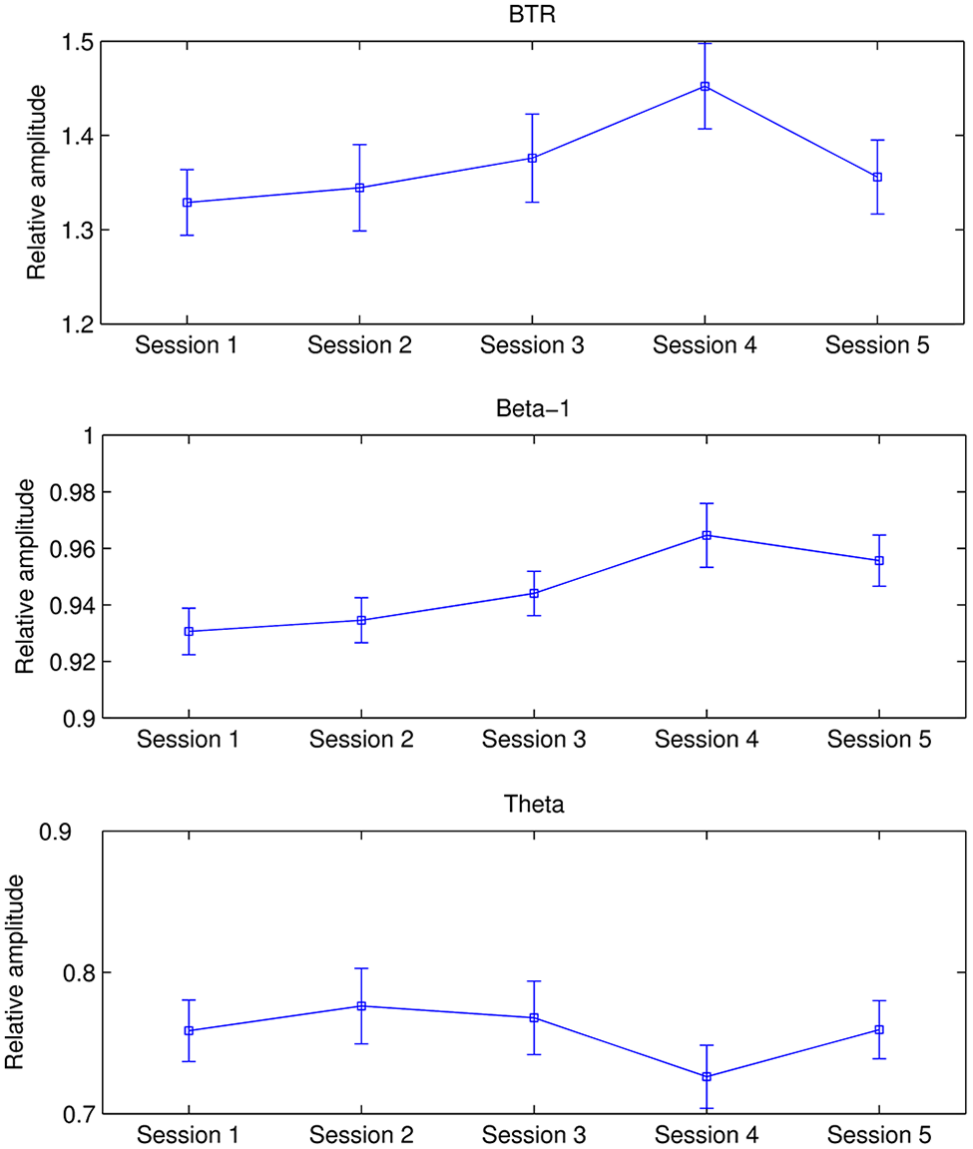
**Mean BTR, beta-1, and theta in each session.** The error bars indicate SEM.

**Figure 2** presents the mean beta-1, theta, and BTR across all participants in each session. As shown in **Figure 2**, BTR increased from Session 1 to Session 4 and then decreased in Session 5. The factor Session showed a significant main effect in BTR [*F*(3.633,323.367) = 3.365, *p* = 0.013, $\eta_{p}^{2}$ = 0.036] and beta-1 [*F*(2.9,258.115) = 4.765, *p* = 0.003, $\eta_{p}^{2}$ = 0.051] but not in theta, alpha and low beta. Further pairwise comparisons with the Bonferroni correction found that BTR in Session 4 was significantly higher than Session 1 (*p* = 0.014), and beta-1 in Session 4 was significantly higher than Session 2 (*p* = 0.012) and marginal significantly higher than Session 1 (*p* = 0.052). Thus, the NF training could increase BTR and beta-1 but not decrease theta across sessions. Moreover, the training did not have influence in alpha and low beta, in accordance with the training independence (Zoefel et al., 2011).

#### NF Learning Prediction

**FIGURE 3:**
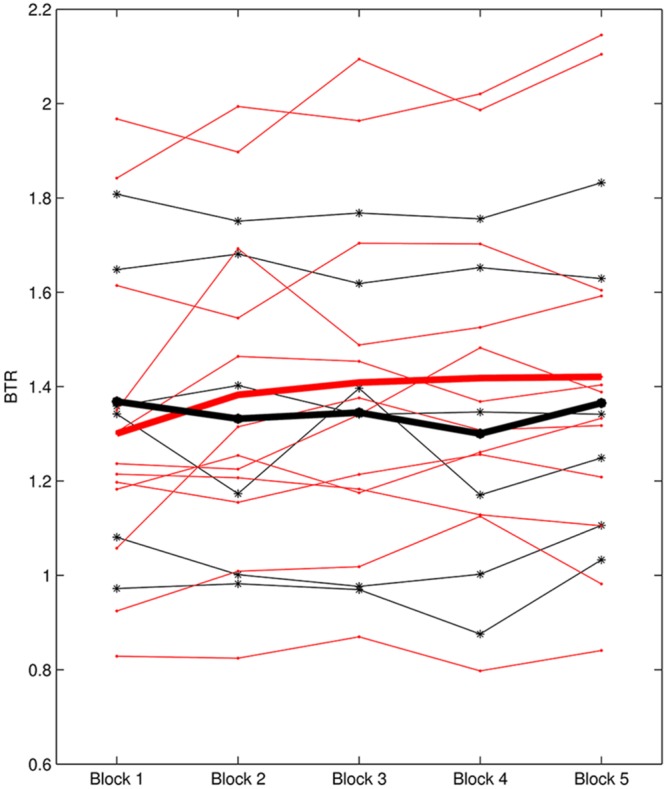
**BTR within sessions of learner_L1 and non-learner_L1.** Thin red lines with dot represent BTR of each learner; thick red line represents the mean BTR across all learners; thin black lines with star show each non-learner; thick black line represents the mean across non-learners.

**FIGURE 4:**
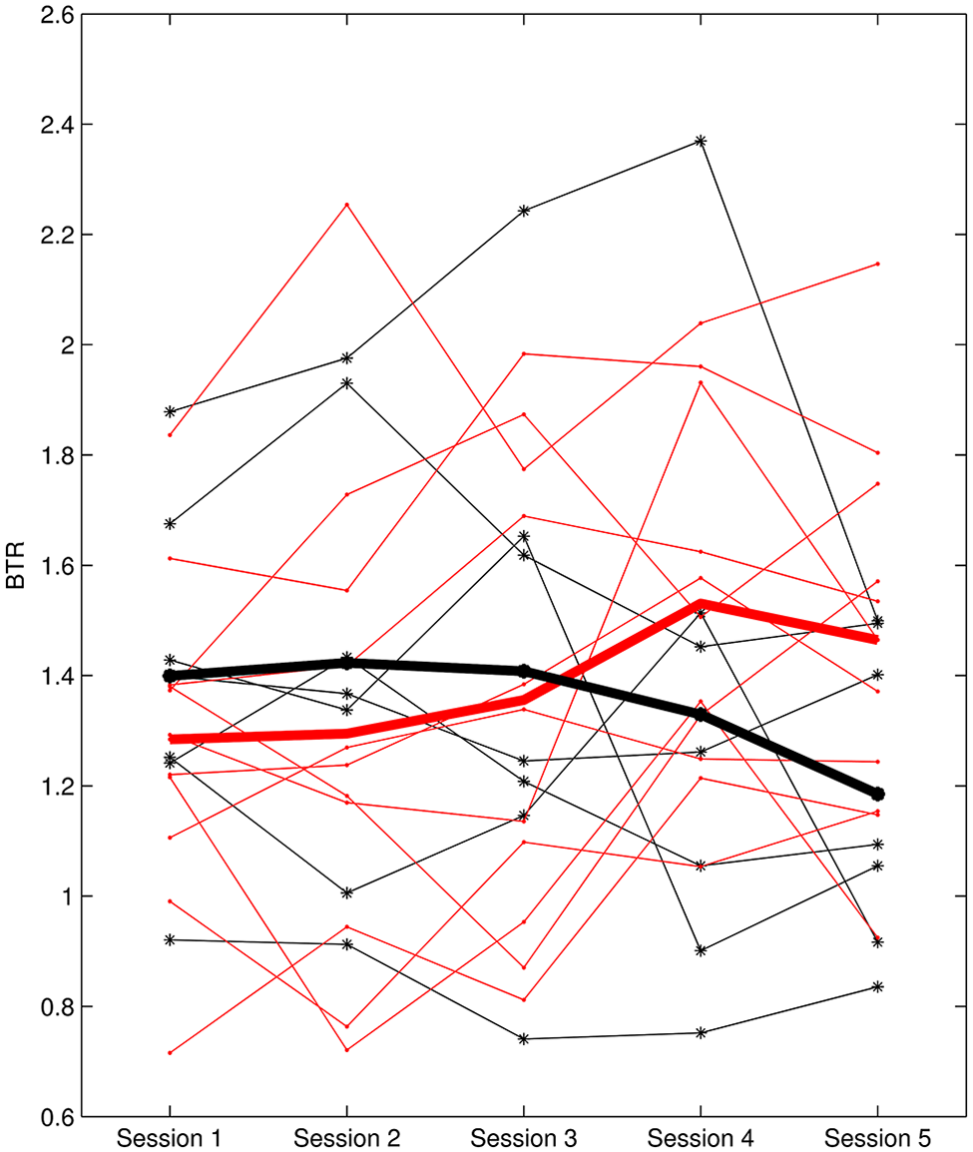
**BTR across sessions of learner_L2 and non-learner_L2.** Thin red lines with dot represent BTR of each learner; thick red line depicts the mean BTR across all learners; thin black lines with star show each non-learner; thick black line shows the mean across non-learners.

L1 ranged from -0.37 to 1.08 and L2 was between -0.118 and 0.111. According to L1, six subjects were identified as non-learners and 12 subjects were learners. On the other hand, seven subjects were non-learners and 11 subjects were learners based on L2 evaluation. **Figure 3** presents the BTR within sessions of learner_L1 and non-learner_L1 and **Figure 4** depicts the BTR across sessions of learner_L2 and non-learner_L2. As shown in the figures, the BTR learning has large inter-individual difference and the trend differences of group mean between learners and non-learners are obvious.

A noteworthy result is that non-learner_L1 was the learner_L2 while non-learner_L2 was the learner_L1. We can see that different evaluation criteria in NF learning may give different learner and non-learner population, but they are not conflicted because of the different NF learning aspects. It seems that the subject who cannot increase BTR across the whole training course would not necessarily fail in increasing BTR within sessions, and vice versa.

There was no significant difference in the examined EEG features between learner_L1 and non-learner_L1. On the contrary, significant differences between learner_L2 and non-learner_L2 were found in low beta at resting baseline with eyes-open [*t*(16) = 2.534, *p* = 0.022] and eyes-closed [*t*(16) = 2.493, *p* = 0.024], and beta-1 in Block 1 of Session 1 [*t*(15) = 3.103, *p* = 0.007]. Due to beta-1 in Block 1 of Session 1 had one outlier, we removed this subject in the above *t*-test and in the following analysis.

The above three significant discriminant features between learner_L2 and non-learner_L2 were taken as input of step-wise LDA. As a result, low beta at eyes-open resting baseline before NF and beta-1 in Block 1 of Session 1 were the predictors to classify learner_L2 and non-learner_L2. Leave-one-out cross-validation revealed that 88.2% of 17 participants could be classified correctly.

## Discussion

The present study employed the BTR training using a bipolar montage of two electrodes directly under electrode sites O1 and O2 and barely above the inion (Hammond, 2005). Although this protocol has shown positive effects in patients with different diseases (Hammond, 2005; Azarpaikan et al., 2014; Sadeghi and Nazari, 2015), the potential effects of this protocol have not yet been fully investigated. Considering the potential of this protocol on treatment of balance problems and enhancement of peak performance (Hammond, 2005) as well as the importance of NF learning prediction, this study aimed to predict the learning ability of this protocol in healthy young adults. To the best of our knowledge, it is the first attempt to apply this protocol to healthy people.

We first examined the NF effects on EEG from the within sessions compared to baseline for the whole NF group. In line with our training objective, BTR obtained a significant increase within sessions compared to baseline. Furthermore, BTR increase mainly resulted from theta decrease because theta revealed a significant decrease but beta-1 only had a slight enhancement. Besides the training band, alpha and low beta bands also showed changes within sessions compared to resting baseline. The increase in beta and decrease in theta and alpha may result from both NF training and high attention in NF. On one hand, NF training is an operant conditioning paradigm which can modulate neuroplasticity by enabling the training individuals to learn to self-regulate their brain activity. In this training, BTR consisted of both beta-1 and theta, and the increase in BTR by NF is certainly associated with the theta decrease. On the other hand, NF training requires subjects to keep attention on the training, whereas the high attention during training compared to the resting state is associated with the increase in beta and the decrease in theta and alpha (Gross et al., 2004; Oken et al., 2006; Fan et al., 2007). Similarly, the broader effect on neighboring bands within sessions was also reported by Ros et al. (2013) in which down-regulation of alpha within session was associated with reductions in theta and beta. Gruzelier (2014b) further pointed out that the NF process itself would call on a range of processes such as learning, attention, motivation, effort, reinforcement monitoring, etc., which may invoke a number of frequency bands.

Although alpha decreased and low beta increased within session, they did not change across sessions. More importantly, consistent with the training objective, BTR showed significant increase across sessions. Furthermore, the neighboring bands result from across sessions is agreement with the training independence proposed by Zoefel et al. (2011) in which upper alpha training had significant effect only on upper alpha band. Likewise, a recent research by Quaedflieg et al. (2015) reported that the asymmetry changes in the right group was independent of other frequency bands in NF training of individual frontal alpha asymmetry. However, some studies also reported the contrary results. For example, alpha NF elicited changes from delta to sigma frequencies (Nan et al., 2012) across sessions, theta NF was associated with additional changes in the alpha and beta frequency across sessions (Enriquez-Geppert et al., 2014b), SMR NF effects extended to a broad beta band (16–25 Hz; Schabus et al., 2014), and gamma (36–44 Hz) NF affected the higher frequency bands from 30 to 60 Hz (Keizer et al., 2010). On the basis of the inconsistent results about across sessions in the literature, it is therefore plausible to assume that the training independence depends on the different training protocols.

By further analysis, this BTR enhancement across sessions was mainly due to beta-1 enhancement across sessions. Interestingly, Hong and Lee (2012) performed NF training to decrease frontal theta/beta ratio in children with intellectual disability, and they found the decline of theta/beta ratio after NF training on account of theta decrease. Thus, ratio training seems complicated and the training results may differ between different subject populations. On the other hand, although the present protocol proposed by Hammond (2005) has shown balance and attention improvement in patients (Hammond, 2005; Azarpaikan et al., 2014; Sadeghi and Nazari, 2015), the EEG change during training was only reported by Azarpaikan et al. (2014) in which the beta-1 and theta were taken as feedback parameter simultaneously. It was found that the Parkinson’s patients could increase beta-1 and decrease theta across eight training sessions (Azarpaikan et al., 2014). The training effects on EEG may vary with different subject population and even in the same subject population the training results had large inter-individual difference.

It should be noted that NF effects on EEG were only examined by within sessions and across sessions in the training location. Some studies have further demonstrated that the NF positive effects on EEG/behavioral performance could be maintained stable at a follow-up of 3-months (van Boxtel et al., 2012; Schmidt and Martin, 2015), 6-months (Leins et al., 2007; Gevensleben et al., 2010; Li et al., 2013; Meisel et al., 2014), 1-year (Weiler et al., 2002), and even 2-years (Becerra et al., 2006; Sürmeli and Ertem, 2011). Kerson et al. (2013) also proposed a NF protocol for ADHD treatment and planed follow-up to 2 years. Thus, our future work would investigate whether the present NF also has some long lasting effects.

A number of studies have shown the large inter-individual difference in NF learning and even non-learners occur in a variety of NF protocols, as mentioned in Section “Introduction.” However, the reason of NF learning difference has been rarely investigated. The control belief and mental activity may play an important role in some training protocols (Nan et al., 2012; Kober et al., 2013; Witte et al., 2013). On the other hand, NF learning may depend on the training protocol since Quaedflieg et al. (2015) found out that the NF learning in frontal alpha asymmetry were dependent on training group, with participants in the right NF group being more likely to change their frontal asymmetry in the desired direction. Besides the NF learning difference, the assessment criteria of NF learning are also heterogeneous as discussed in recent studies (Gruzelier, 2014b; Wan et al., 2014; Reichert et al., 2015; Zuberer et al., 2015). Some studies assess the NF learning by the difference of training parameter between the last session and the baseline before training (e.g., Zoefel et al., 2011), between the first session and the last session (e.g., Dekker et al., 2014), between the average of the first two sessions and the average of the last two sessions (e.g., Studer et al., 2014), or between two resting baseline (e.g., Quaedflieg et al., 2015). On the other hand, the NF learning has been also assessed by the training parameter changes within sessions (e.g., Ros et al., 2009; Enriquez-Geppert et al., 2013; Wan et al., 2014; Reichert et al., 2015) or across sessions (e.g., Ros et al., 2009; Enriquez-Geppert et al., 2013; Kouijzer et al., 2013; Wan et al., 2014). Furthermore, some studies utilized more than one criterion to evaluate the learning ability (e.g., Ros et al., 2009; Weber et al., 2011; Enriquez-Geppert et al., 2013). Gruzelier (2014b) concluded that it would be helpful always to report learning functions within sessions, across sessions and with successive baselines in order to understand the NF processes. Zuberer et al. (2015) also suggested that it might be interesting to include within session analyses or cross session changes respectively. Furthermore, our previous work in the prediction of alpha NF learning found that both across session and within session learning could be predicted by the same predictor (i.e., resting alpha amplitude; Wan et al., 2014). As a consequence, this study assessed the BTR NF learning from both within sessions and across sessions, respectively.

As stated by Gruzelier (2014b), it might be better to use an early training performance as the baseline, which would offer the participant a sense of achievement. Thus, the NF learning within sessions (i.e., L1) utilized the changes of later blocks compared to Block 1, in which Block 1 was taken as a type of baseline. A positive BTR value in later blocks compared to Block 1 was expected, indicating that the participant could increase BTR within sessions (i.e., Learner_L1). Regarding the NF learning across sessions (L2), a positive linear slope between BTR and session number was desired, suggesting that the participant could enhance BTR across sessions (i.e., Learner_L2); six non-learner_L1 and seven non_learner_L2 were found in a total of 18 participants. It is very interesting that even for the same participant, the learner identification differed between learning evaluation criteria. In this study, non-learner identified by L1 was the learner determined by L2 while non-learner determined by L2 was the learner assessed by L1. These results are not contradictory, because L1 expressed the learning ability in short time while L2 focused on the accumulative NF learning in long term. From the different learner definitions, the subject who could not increase BTR within sessions may be able to keep BTR increase across whole training procedure, and vice versa.

We did not find predictor to predict learner and non-learner based on L1, but it is not the case for L2. Low beta at resting baseline with eyes-open and eyes-closed as well as beta-1 in Block 1 of Session 1 was significant higher in learner_L2 than non-learner_L2. More importantly, we found that low beta at eyes-open resting baseline and beta-1 in Block 1 of Session 1 could predict learners and non-learners evaluated by L2. The resting and initial beta amplitudes as predictors of learning ability in BTR NF were in accordance with the previous findings from other training protocols. For instance, resting alpha amplitude predicted the NF learning across sessions in alpha NF (Wan et al., 2014) and resting SMR power predicts the NF learning within sessions in SMR NF (Reichert et al., 2015), and Enriquez-Geppert et al. (2013) demonstrated a significant positive correlation in the training performance between Session 2 and the last session in theta NF. Our result indicates that only a 1-min eyes-open resting baseline and one training block with 4.5 min duration could predict the learning ability across the whole training procedure, which reveals a convenient and low cost way for NF learning prediction.

Apart from the EEG predictors, the morphology of brain structures as predictors of NF learning was reported in two recent studies as well. More specifically, Enriquez-Geppert et al. (2013) found that volume of the midcingulate cortex as well as volume and concentration of the underlying white matter structures predicted the NF learning within sessions in up-regulation of frontal-midline theta NF. Likewise, a recent research demonstrated that the NF learning within sessions in up-regulation SMR training was predicted by the volumes in the anterior insula bilaterally, left thalamus, right frontal operculum, right putamen, right middle frontal gyrus, and right lingual gyrus, while the gray matter volumes in the supplementary motor area and left middle frontal gyrus predicted the NF learning in up-regulation gamma training (Ninaus et al., 2015). These findings inspired us to examine the morphology of brain structures in further BTR NF study.

The present study is limited by lack of control group. Future research should include an appropriate sham-NF control group to extend the validity of current results. Additionally, cognitive performance and behavioral measurement will be added in order to explore the benefits of this training protocol in healthy people. What’s more, the training effects on the behavioral performance between learners and non-learners will be analyzed in future work.

To summarize, we demonstrated that low beta in 1-min eyes-open resting state before NF and beta-1 in the first training block with 4.5 min could predict the BTR learning across sessions, providing a low cost, convenient and easy way to predict the BTR NF learning. It is helpful to prevent the potential frustration of non-learners, adjust the NF protocol accordingly and understand the neural mechanisms of this training protocol. It should be notable that this study was based on the healthy people and used bipolar montage directly under electrodes sites O1 and O2. Whether the BTR NF in patients and with different training locations shares the same EEG predictors also deserves more investigation.

## Conflict of Interest Statement

The authors declare that the research was conducted in the absence of any commercial or financial relationships that could be construed as a potential conflict of interest.
